# Computational Analysis of the Ligand Binding Site of the Extracellular ATP Receptor, DORN1

**DOI:** 10.1371/journal.pone.0161894

**Published:** 2016-09-01

**Authors:** Cuong The Nguyen, Kiwamu Tanaka, Yangrong Cao, Sung-Hwan Cho, Dong Xu, Gary Stacey

**Affiliations:** 1 Division of Plant Sciences and Biochemistry, Christopher S. Bond Life Sciences Center, University of Missouri Columbia, Missouri, 65211, United States of America; 2 Department of Plant Pathology, Washington State University, Pullman, Washington, 646430, United States of America; 3 Department of Computer Science, Informatics Institute, Christopher S. Bond Life Sciences Center, University of Missouri, Columbia, Missouri, 65211, United States of America; Griffith University, AUSTRALIA

## Abstract

DORN1 (also known as P2K1) is a plant receptor for extracellular ATP, which belongs to a large gene family of legume-type (L-type) lectin receptor kinases. Extracellular ATP binds to DORN1 with strong affinity through its lectin domain, and the binding triggers a variety of intracellular activities in response to biotic and abiotic stresses. However, information on the tertiary structure of the ligand binding site of DORN1is lacking, which hampers efforts to fully elucidate the mechanism of receptor action. Available data of the crystal structures from more than 50 L-type lectins enable us to perform an *in silico* study of molecular interaction between DORN1 and ATP. In this study, we employed a computational approach to develop a tertiary structure model of the DORN1 lectin domain. A blind docking analysis demonstrated that ATP binds to a cavity made by four loops (defined as loops A B, C and D) of the DORN1 lectin domain with high affinity. *In silico* target docking of ATP to the DORN1 binding site predicted interaction with 12 residues, located on the four loops, via hydrogen bonds and hydrophobic interactions. The ATP binding pocket is structurally similar in location to the carbohydrate binding pocket of the canonical L-type lectins. However, four of the residues predicted to interact with ATP are not conserved between DORN1 and the other carbohydrate-binding lectins, suggesting that diversifying selection acting on these key residues may have led to the ATP binding activity of DORN1. The *in silico* model was validated by *in vitro* ATP binding assays using the purified extracellular lectin domain of wild-type DORN1, as well as mutated DORN1 lacking key ATP binding residues.

## Introduction

The adenine 5'-tri-phosphate (ATP) is an essential, energy-rich molecule found in all living cells. ATP not only serves as a key substrate and co-factor in a variety of intracellular biochemical reactions and signaling processes, but also can be exported to the extracellular matrix where it serves as an intercellular signaling molecule [1–5]. In plants, the extracellular release of ATP can be triggered by touch [6,7], wounding [8], biotic [9–11] and abiotic stresses [6,12], and during normal growth and development [13–16]. In both plants and animals, recognition of extracellular ATP (eATP) triggers an increase in cytoplasmic Ca^2+^ influx, increase in reactive oxygen species (ROS), elevation of nitric oxide levels and specific gene expression [17,18]. The recognition of eATP in animals is mediated by two classes of purinoreceptors, P2X and P2Y [19]. Activation of P2X receptors gates ion flux, while activation of P2Y receptors recruits heteromeric G proteins that activate other intracellular processes. In contrast, plants appear to lack both P2X and P2Y receptors. Therefore, there was considerable interest in the recent elucidation of the first plant purinoreceptor, DORN1 (DOes not Respond to Nucleotides 1), which represents a new, kinase family of purinoreceptors P2K (Purinergic 2K receptors, K is for kinase) [17]. DORN1 is a typical receptor-like protein kinase, with an N-terminal signal peptide, an extracellular L (legume)-type lectin domain, a transmembrane domain, and a C-terminal, active kinase domain. The lectin domain of DORN1 shares remarkable sequence similarity to other L-type lectins; that is, 30 to 35% sequence identity or 47.5% to 52.7% sequence similarity to well-studied L-type lectins. In addition, secondary structural prediction suggests a significant resemblance in the folding pattern of DORN1 relative to other L-type lectins [20].

L-type lectins bind carbohydrates reversibly and specifically without enzymatic modification of the carbohydrate. L-type lectins are ubiquitous in land plants and they represent the largest and most thorough studied group among other lectin families [21,22]. Currently, 248 tertiary structures and quaternary complexes of 54 L-type lectins, with or without carbohydrates and other small ligands, have been crystallized and deposited into the Protein Data Bank (PDB) [23]. Most of these lectins dimerize or tetramerize to form homomeric or heteromeric complexes. The tertiary structures of L-type lectin subunits or protomers are in the shape of a dome, and they are almost superimposable. The protomers are made of a relatively flat six-stranded β-sheet (called back face) and a curved seven-stranded β-sheet (called front face), interconnected by β-turns and loops. The strands run back and forth between the front and back sheets, and anti-parallel to each other within each sheet, therefore strengthening and stabilizing the core structure [21]. Each L-type lectin harbors a carbohydrate combining site, and two adjacent conserved metal binding sites for Ca^2+^ and Mn^2+^ [21,22]. These divalent cations are essential and play important roles in carbohydrate interaction [24]. In general, the carbohydrate binding site is structurally conserved among L-type lectins and consists of residues from four separate loops A, B, C and D located at the top of the dome. Sequence comparison of a number of L-type lectins displays a variability not only in sequence conservation but also in size of the four binding loops, which likely define the carbohydrate binding specificity and affinity for each lectin. However, several key binding residues in the respective four loops are highly conserved [25]. Many L-type lectins, but not all, are post-translationally modified by glycosylation and possess at least one to several glycan chains of either the high-mannose or complex type [26,27]. Glycosylation of L-type lectins often results in the formation of the quaternary complexes of L-type lectins, either stabilizing or destabilizing the non-covalent association of the subunits to form the dimeric assembly or preventing the formation of the tetrameric assembly from the dimers, respectively [28]. So far, there is no evidence that the glycan chains play any structural role in ligand binding by L-type lectins.

In this study, in order to further our understanding of DORN1 function, we combined and integrated various *in silico* approaches to predict the DORN1 eATP binding pocket and to identify the key amino acids involved in binding. The predictions were then validated by *in vitro* studies using the purified, wild-type DORN1 ectodomain, as well as those from mutant proteins lacking key eATP binding residues. Our results give insight into the molecular interactions between DORN1 and eATP, providing useful information for further studies of plant, extracellular nucleotide receptors.

## Materials and Methods

### *In silico* design for three dimensional (3D) modeling, binding site prediction and ligand docking of eATP to the DORN1 L-type lectin domain

**Fig 1:**
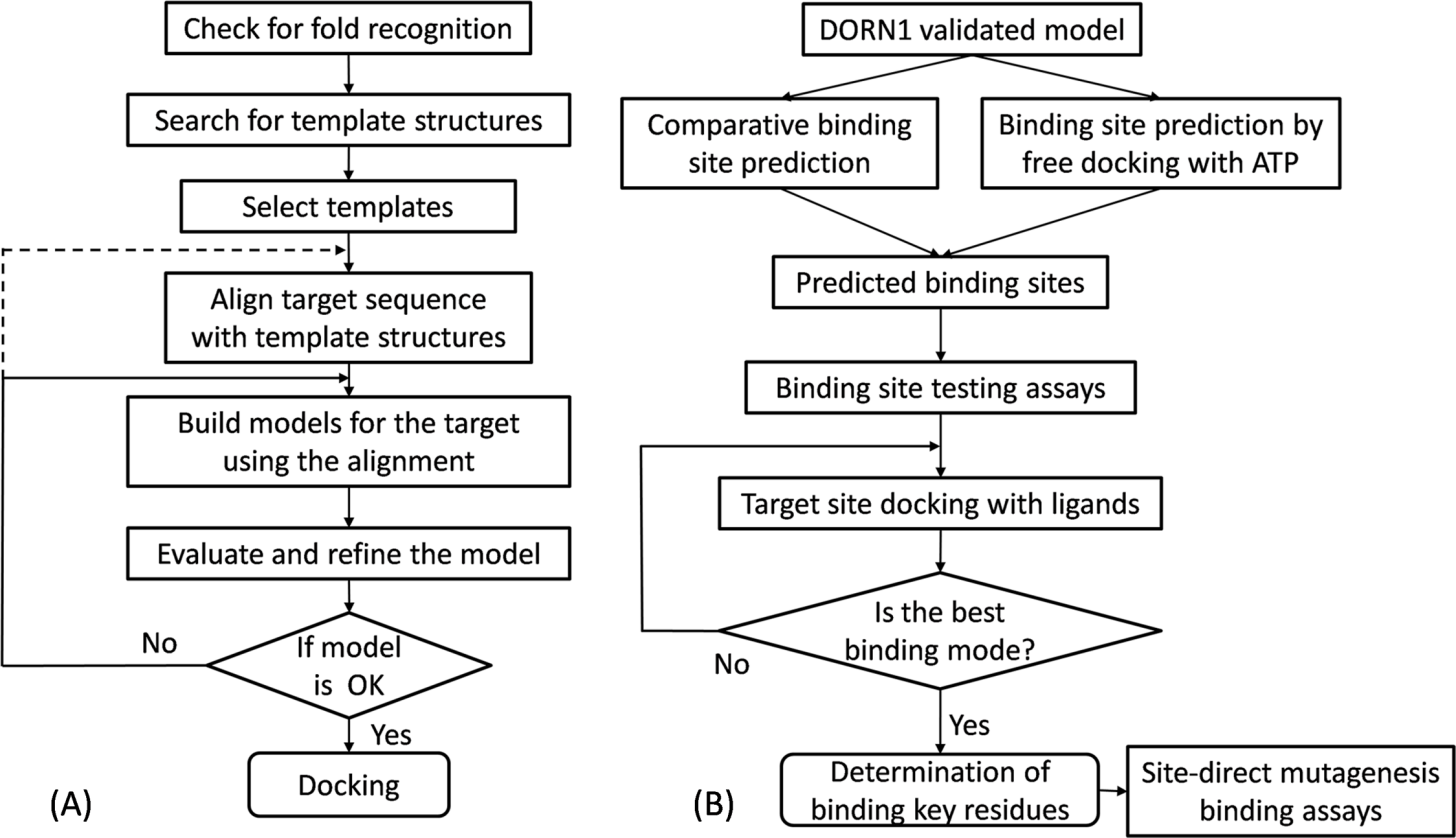
Workflow for homology modeling and molecular docking of the DORN1 L-type lectin domain. The molecular modeling of DORN1 consists of two major prediction processes, (A) comparative modeling and (B) protein-ligand docking. Homology modeling of DORN1 involves fold assignment, template search, target-template alignment, model generation, and model evaluation and validation. DORN1-ligand docking includes energy minimization with molecular dynamic simulation, binding site prediction, and DORN1-ligand docking and analysis.

*In silico* experimental design and the work flowcharts for 3D modeling and binding site prediction of the lectin domain of DORN1 are shown in Fig 1A and 1B. In brief, the sequence of the lectin domain of DORN1 was obtained from the UniProt database [29] and first checked for its folding topology. This information was used to search for structurally similar candidates in the PDB. Once good template candidates were identified, the protein sequence of the DORN1 lectin domain was aligned with their crystal structures. Initially, a pool of 3D models of the DORN1 lectin domain was created using a homology modeling approach. Subsequently, a number of best energy ranked models were chosen for the evaluation of their stereochemistry properties (Fig 1A). The top quality 3D model was selected and optimized with molecular dynamic simulation. The optimized 3D model was utilized to predict theATP binding site using either free docking of ATP to the 3D model or template-based binding site prediction tools. After the candidate binding site was identified by docking and confirmed by direct-binding assays, target docking of ATP to the confirmed site was performed by superimposing a binding grid cover the entire site. The ATP-DORN1 binding complex was analyzed to retrieve the best binding mode and key interacting residues (Fig 1B).

### Folding recognition and template search

The sequence of 236 amino acids of the DORN1 lectin domain was retrieved from the UniProtKB database (entry code Q9LSR8). The sequence was fed into the FUGUE server tool [30] for fold topology confirmation of the DORN1 extracellular domain in order to confirm the overall structural fold similarity to that of crystallized L-type lectins. The next step used the same sequence to search against the PDB for templates by the HHSEARCH online tool [31]. Candidate templates were selected based on three major criteria: high percentage of sequence identity, crystal structural quality (low resolution), and sequence coverage in the alignment.

### Alignment, homology modeling and quality assessment of DORN1 models

Alignment is an important procedure in homology modeling which determines the quality of a target structure. Aligning a target sequence to template structures provides better models than using template sequences because protein structure is more conserved than sequence [32]. The crystal structures of the chosen templates were downloaded from the PDB and filtered to retain only monomer structures. Each monomer was selected from complexes that had no missing residues as denoted by a chain number after the structural identifier (Table 1). The ectodomain sequence (236 amino acids) of DORN1 was aligned with the crystal structure of the templates using the Align2D module of the Modeller (version 9.14) [33] and then manually checked to ensure a proper alignment, which influenced the quality of the 3-D models. Since the specific metal binding sites are well resolved structures and highly conserved, two divalent cationsCa^2+^ and Mn^2+^ of the 3IPV (SPL) structure were also added in the alignment, which allowed these cations to be included in the DORN1 model.

**Table 1:** Search statistics of three selected legume lectin templates used for homology modeling of the DORN1 L-type lectin domain.

PDB ID^1^	Identity & similarity (%)^2^	Resolution (Å)	Aligned columns	E-value	Identified ligands	Complex
1BJQ (f)	30 (47.3)	2.50	25–253	3.7E^-56^	Multiple^3^	Octamer
1FAT (a)	32 (52.2)	1.75	23–250	1.5E^-56^	Mannose	Tetramer
3IPV (a)	35 (50.5)	2.04	2–235	4.5E^-53^	Galactose	Tetramer

^1^ Protein code in the PDB starts with a number and follows by letters or numbers, letter in round brackets is chain number.

^2^ Number in round brackets indicates same groups of amino acids.

^3^ High preference for N-Acetylgalactosamine over galactose.

From the alignment, a pool of 2000 models for DORN1 was generated using the Model-ligand module of the Modeller. A reasonable model was selected and analyzed based on the Modeller’s probability density function with a low discrete optimized protein energy score (DOPE). Again, the residue-by-residue and overall structural geometry of the model was checked using the Verify3D web server [34] and the Ramachandran plot (Laskowski *et al*., 1993) embedded in the Rampage web server [35]. If any residue(s) in the model was detected to have a ‘bad’ geometry (i.e., ‘residues in outlier regions’), the model was repeatedly refined using the Loop refine module of the Modeller until no residue was found in the outlier regions based on the Ramachandran plot. In addition, the best 3D structure of the model was checked against its templates by superimposing their structures and calculating the root mean square difference (RMSD) values among their corresponding residues.

### Molecular dynamic simulation

Initially, the DORN1 model was optimized by Modeller. However, we found that the model could be further improved using molecular dynamic (MD) simulation. We performed the MD simulation to optimize the DORN1 structure prior to the docking experiments. The refined model was subjected to MD simulations for energy minimization of side chains, keeping the rigid backbone. The MD calculation used the all-atom CHARMM22 protein force field [36] at a constant temperature of 300 K and a pressure of 1 bar, using the method described by Pedretti [37]. The model was checked, fixed for errors and coordinates normalized using the VegaZZ tool [37]. The charge and hydrogen atoms were added to the target structure and additional CHARMM22 parameters for the VegaZZ were also generated. Finally, NAMD version 2.10 (NAno scale Molecular Dynamics program) [38] embedded in the VegaZZ tool was used to perform the MD simulation on the target model. MD results were generated using the VMD-Visual Molecular Dynamic tool [39]. We also attempted to perform an MD simulation using a flexible backbone, but the whole structure was distorted when superposed on the template structures and it could not be utilized for the docking experiments.

### Identification of ligand binding sites

The model obtained in the MD simulation step was used for ligand binding site predictions. The eATP binding pocket was predicted using either the blind docking method or online binding site prediction tools. In blind docking, a large grid box was created on the entire surface of the target protein structure for the screening and detection of possible binding pockets and binding modes of the ligand (S1A Fig). The blind docking experiments used the MGL tool (version 1.5.6) [40] and the docking algorithm embedded in the AutoDockVina tool, a popular and free docking tool [41]. The online tools predicted an eATP binding pocket based on template sequences and structures, pre-calculated pocket sizes and physicochemical properties of the model. These online prediction tools included COACH [42], COFACTOR [43], FINDSITE [44], and TM-Site [42]. Binding sites obtained from these methods were combined and filtered, and the putative sites were tested using *in vitro* ATP binding assays for site-directed mutagenesis. Target dockings were then performed on the site confirmed by the direct-binding assay to predict key interacting residues.

### *In vitro* ATP binding assay for site-directed mutagenesis

**Fig 2:**
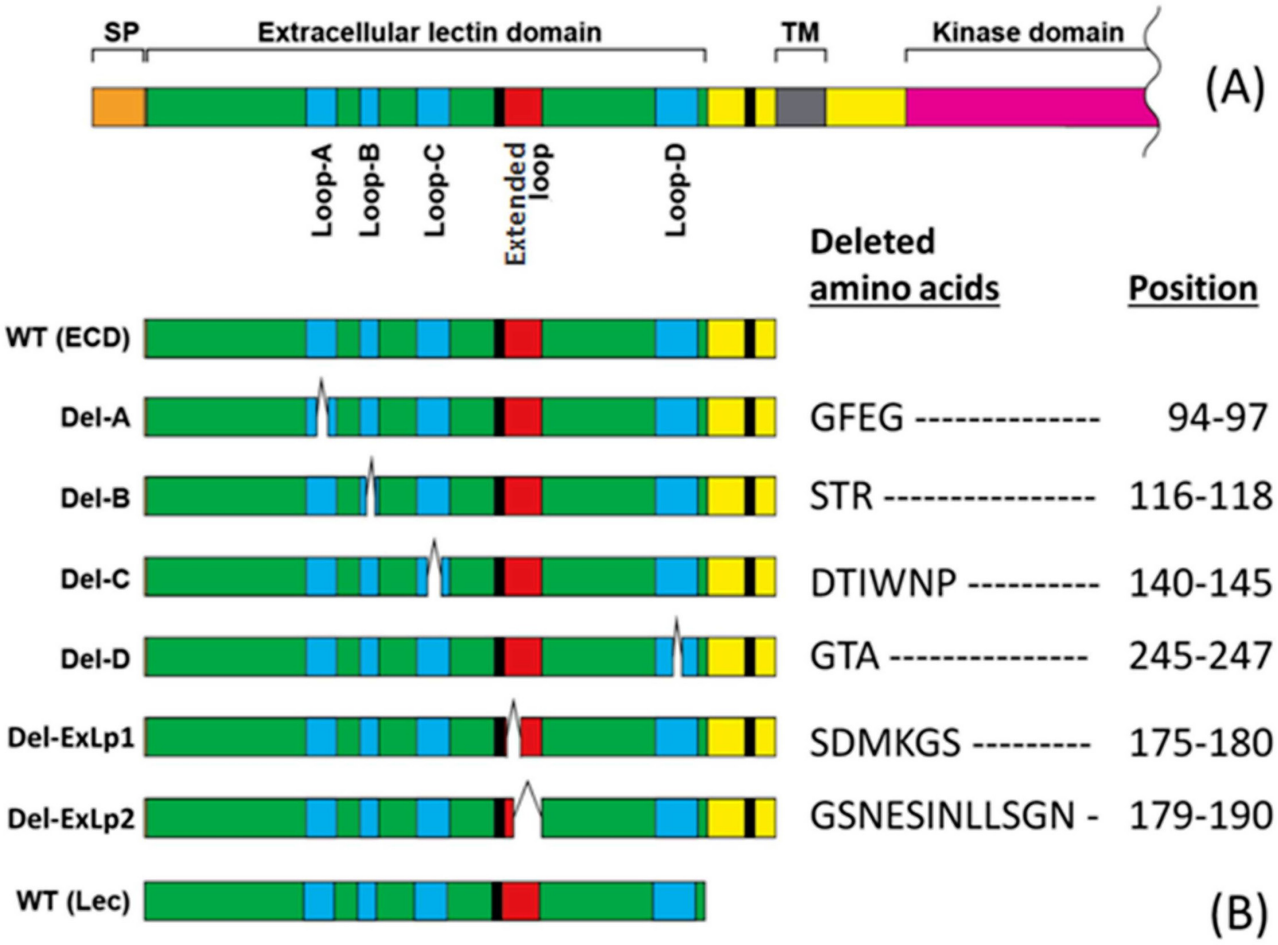
Schematic representation and loop deletion information of DORN1 for site-directed mutagenesis binding assays. (A) A schematic representation drawn into scale and sequence features of the DORN1 protein. (B) Detailed information of loop deletion mutations (white bars) on ATP binding activity of DORN1, WT (ECD): wild-type extracellular domain, Del-A: loop A deletion, Del-B: loop B deletion, Del-C: loop B deletion, Del-D: loop D deletion, Del-ExLp1: deletion of extended loop 1, Del-ExLp2: deletion of extended loop 2, WT (Lec): wild-type lectin domain.

In this study, our primary purpose is to reveal DORN1’s structural contribution to the ligand binding. Previous binding studies between legume lectins and carbohydrate revealed that the length of four binding loops varies among lectins [25]. Furthermore, both the loop B and loop C are critical for their binding activity. Therefore, we tested loop deletions for the ATP binding activity of DORN1. Initial *in vitro* binding assays were conducted to confirm the importance of identified binding loops since there were two possible ATP binding sites obtained from the *in silico* predictions. Detailed information of DORN1 linear structure and DORN1 mutant proteins is provided in Fig 2A and 2B, respectively.

Briefly, the extracellular domain of DORN1 was PCR amplified with a gene specific primer set: 5’-CGCGGATCCAACAAGAGACAAGCTTTGTCTAT-3’ and 5’-TTCCTCGAGTTATGTAGATACTTTCTTATGTGGAGC-3’. The PCR product was digested with BamHI and XhoI and cloned into the pGEX-5X-GST vector (GE Healthcare). This clone was used as a template for *Dpn*I-mediated site-directed mutagenesis using the following primer set: 5'-GTGCTTTGGTGCCTAAGCCAGGCCATGGTATTGTCTTTGT-3' and 5'-ACAAAGACAATACCATGGCCTGGCTTAGGCACCAAAGCAC-3' for the loop A deletion (GFEG deletion); 5'-ATGGACTTTACTCACGCAGAATACTTGGGGATTTTCAATGCTTC-3' and 5'-GAAGCATTGAAAATCCCCAAGTATTCTGCGTGAGTAAAGTCCAT-3' for the loop B (STR deletion); 5'-CACGTACTTGCTGTTGAGCTTGATTTCAAAGATATTGACCAC-3' and 5'-GTGGTCAATATCTTTGAAATCAAGCTCAACAGCAAGTACGTG-3' for Loop-C (DTIWNP deletion); 5'-GGTTCTCTGCAGCAACAGGGAGTGATCAATATATTCTCTG-3' and 5'-CAGAGAATATATTGATCACTCCCTGTTGCTGCAGAGAACC-3' the loop D (GTA deletion); 5'-ATAGCTTCAGCATCTTACTATAATGAAAGCATAAACCTCTTG-3' and 5'-CAAGAGGTTTATGCTTTCATTATAGTAAGATGCTGAAGCTAT-3' for the extended loop 1 deletion (SDMKGS deletion); and 5'-CTTACTATTCCGACATGAAACCTATACAGGTCTGGGTGGAT-3' and 5'-ATCCACCCAGACCTGTATAGGTTTCATGTCGGAATAGTAAG-3' for the extended loop 2 deletion (GSNESINLLSGN deletion). The recombinant proteins were isolated after expression in *E*. *coli* strain BL21-AI (Invitrogen) and purified using Glutathione Sepharose 4B (GE Healthcare) beads.

For the *in vitro* eATP binding assays, the purified proteins were incubated on ice for 30 minutes with [γ-32P] ATP (PerkinElmer; specific activity 800 Ci mmol-1) in a 100 μl reaction mixture containing 10 mM HEPES (pH 7.5) and 5 mM MgCl_2_ with the presence or absence of 100-fold unlabeled ATP (to define specific binding) or other nucleotides (for the ligand competitive binding assays). Note that comparable experiments using [α-32P] ATP gave similar results. Unbound nucleotides were separated from the protein by passage through a Sephadex PD MiniTrapG-25 gel filtration column (GEHealthcare). Bound radio ligand was collected into scintillation vials and mixed with scintillation cocktail (MP Biomedicals). The radioactivity was quantified by liquid scintillation counting (Tri-Carb 2810TR, PerkinElmer). Specific binding of the ligand was estimated as the difference between total binding and non-specific binding. The binding data were analyzed and plotted using a non-linear regression model, equation Y = Bmax*X/(Kd+X) in the R-DRC package (version 2.5–12) of the R tool. Here, Bmax is maximum specific binding extrapolated to very high concentration of radioligand, and Bmax and Kd is the equilibrium binding constant, i.e. the radioligand concentration required to obtain a half-maximal binding at equilibrium.

### Template redocking experiments

Redocking of native ligands, including N-acetyl glucosamine (GalNAc) and Forssman disaccharide (GalNAc(α1–3)GalNAc) and docking of non-native ligands, including ATP, galactose and lactose to one of the legume lectin templates (DBL or 1BJQ) was performed using the AutoDockVina algorithm to validate the consistence of the docking tool and to reveal *in silico* binding energies for comparison between native ligands and non-native ligands. The native ligands were separated from the crystal complex, added with parameters (e.g. polar hydrogen) and given flexible bonds for docking. A similar docking procedure was applied for redocking of native and non-native ligands to DBL (1BJQ) as described in the target docking experiments. The average root mean square deviation (RMSD) of the re-docking and native ligands was calculated using their coordinate data. If the computed RMSD value was less than 2.0Å for the best-scored conformation [45], the docking was considered successful.

### Target docking experiments

The target docking experiments were performed with semi-flexible methods (S1B Fig), i.e. the ligand structures were given flexible conformations for all possible rotatable bonds, while the receptor model was kept rigid. The 3D conformers of 8 nucleotides, galactose (a mono-saccharide), and lactose (a di-saccharide) ligands were obtained from the NCBI Pubchem database and used to dock into the putative binding site of the DORN1model. Supplement dockings of the DORN1 model with other nucleotides, mono- and disaccharides were done to determine the relative binding affinity of different ligand types compared to that of ATP. Before docking, the model and the ligands were prepared using the MGLTools-1.5.6 software [40] to satisfy docking requirements; such as, addition of polar hydrogen atoms, calculation of partial charge using the AMBER force field [36], selection of flexible bonds for the ligand, and adjustment of docking position and grid space. The docking experiments were performed with the AutoDockVina algorithm [41]. The docking models with the lowest binding energy (expressed in kcal/mol) were selected and visualized in the Chimera software [46]. Detailed interaction maps between the ligands and surrounding residues were generated by the LigPlus software [47]. Important residues were considered to guide further deletion and site-directed mutation experiments based on the number of shared hydrogen bonds and hydrophobic interactions.

## Results

### Fold topology, template search and sequence-structure alignment

In previous studies, the extracellular domain of DORN1 was shown to have sequence similarity to L-type lectins [48]. The Hidden Markov Model (HMM) search of the DORN1 ectodomain against the PFAM database confirmed that the ectodomain of DORN1 was from the L-type lectin family with a PFAM identifier of PF00139. The ectodomain sequence of DORN1 was again checked for its fold architecture to reconfirm predictions and classification. Indeed, the top hit topology was the lectin family at a very high Z-score (29.06) and with a significant confidence level as compared to that of other related protein families (S1 Table).

We used the HMM–HMM comparison tool [31] to search for the DORN1 ectodomain sequence against the structure database (PDB) and identified 14 L-type lectins with highly conserved secondary structures. Their pairwise aligned sequences with the DORN1 ectodomain showed the proportion of identical amino acids ranging from 30 to 35% (data not shown). The closest structure with homology to the DORN1 ectodomain was from *Spatholobus parviflorus* (SPL, PDB code 3IPV), which shares 35% identity and 50.5% amino acid similarity in a pairwise alignment (Table 1).

Among the 14 closest homologs, we selected the X-ray crystal structures of three representative templates with the PDB codes 1FAT [49], 1BJQ [50] and 3IPV [51] for a multiple sequence-structure alignment with the DORN1 lectin domain sequence. These templates share significant sequence identity and similarity, low structural resolution and high global sequence coverage over the other L-type lectin homologs (Table 2).

**Table 2:** List of DORN1 interacting residues with ATP obtained from prediction and target docking.

Loop	Knowledge based predictions^1^	Target docking^2^
A	Gly98, His99	Glu96 (1H), Gly98, His99 (2H)
B	Thr117, Arg118	Thr117 (2H), Arg118 (2H)
C	Ile143, Asn145	Ile143, Trp144 (1H), Pro146
D	Gly245, Thr246, Ala247	Gly245, Thr246 (3H), Ala247 (1H)
Total residues	9	11

^1^ Consensus residues were predicted by using different online prediction tools, numbers next to residue names represent actual positions in the full length sequence of DORN1.

^2^ Residues follow by a number and a letter H within round-brackets denote number of hydrogen bonds sharing with ATP, other residues without a number and a letter are those only interact with ATP through hydrophobic interaction.

**Fig 3:**
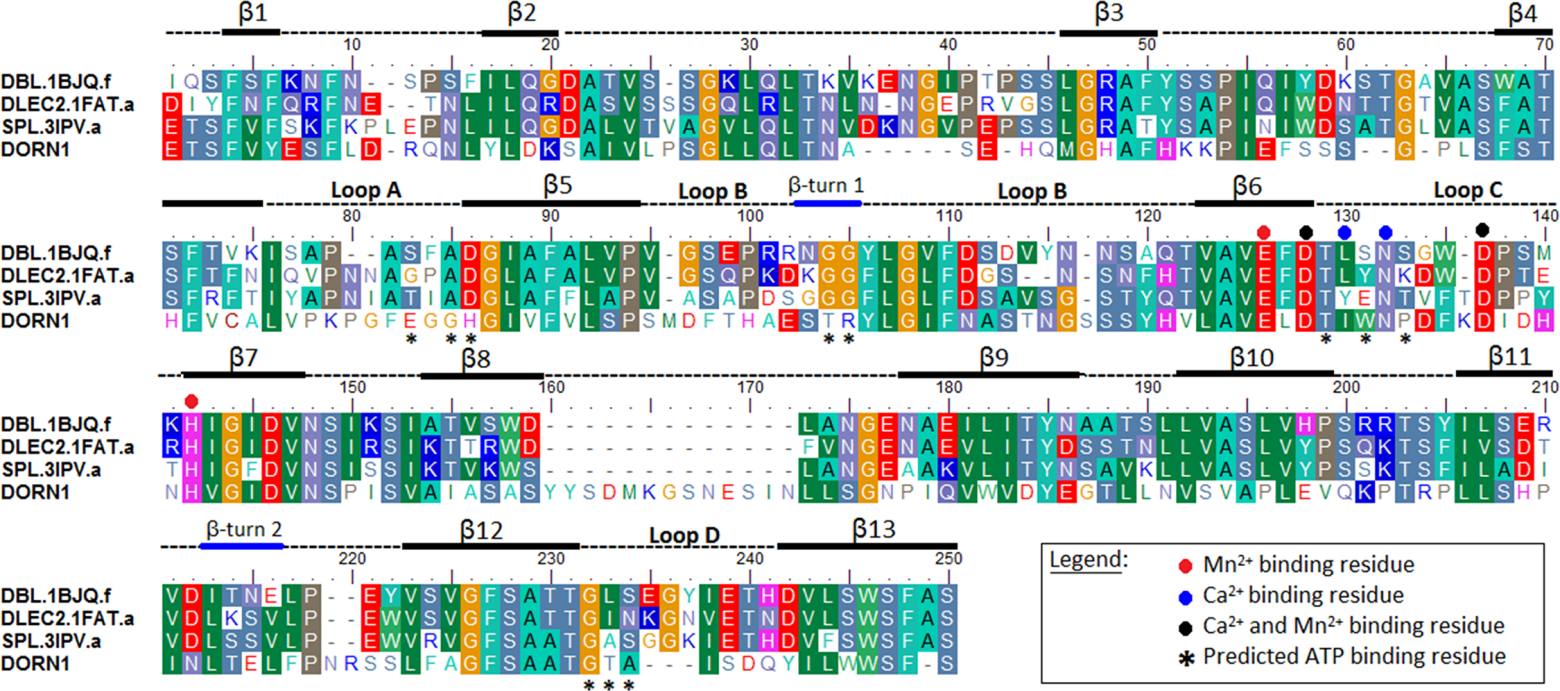
Multiple sequence-structural alignment of the DORN1 L-type lectin domain and three selected templates. Template sequence names were assigned by the Uniprot database, following by a 4-letter PDB code and chain name, separated by a dot. DBL-*Dolichos biflorus* (1BJQ*)*, DLEC2*-Phaseolus vulgaris* (1FAT*)*, SPL*-Spatholobus parviflorus* (3IPV), and DORN1*-Arabidopsis thaliana* (P2K1*)*. Numbers at the beginning and the end of the alignment denote actual amino acid position in the protein sequences. Black bars-beta strands, blue bars-beta turns, dash lines-loops or coils, * predicted ATP binding residue, + conserved sugar binding residue inferred from legume lectins, red dots-Mn^2+^ binding residues, blue dots-Ca^2+^ binding residues, black dots-Mn^2+^ and Ca^2+^ binding residues. Columns with same color indicate identical amino acids or similar groups of amino acids, dashes are gap insertions. The black bars (beta strands) and blue bars (beta turns) below the alignment represent the secondary structure of the DORN1 L-type lectin domain inferred from the modeled DORN1 structure. Round dots with different colors below the aligned columns are cation binding residues inferred from the templates. Black stars denoted predicted ATP binding residues of the DORN1. Loops A, B, C and D and an extended loop inferred from the alignment with legume lectins.

Once the templates were chosen, a sequence—structural alignment was constructed for homology modeling of the DORN1 domain (residues 24–256). From the pairwise alignments of the template search results (the alignment not shown) and the sequence-structure alignments, it was clear that the lectin domain of DORN1 has an insertion of 13 residues between 2 beta-strands, β8 and β9 (the alignment in Fig 3). This insertion, is only found in the family of lectin receptor kinases in the *Brassica* family, and, hence, likely occurred when this plant family diverged from a common ancestor with other angiosperms.

The significance of this extended loop is unknown but it is interesting to note that it contains the ASYY motif, previously shown to be a arginine-glycine-aspartate (RGD) binding domain, which was predicted to play an important role in the ability of DORN1 (LecRK I.9) to mediate connections between the plasma membrane and plant cell wall [20]. The sequence—structural alignment of DORN1 with the L-type lectin templates also showed that most of the conserved residues are located within the beta-strands, including the Ca^2+^ and Mn^2+^ binding sites. In contrast, the intervening loops display significant variability, not only in sequence but also in size. There are deletions or insertions of residues in most of the loops, including four signature loops (A-C) of L-type lectins, which form a carbohydrate binding pocket in the canonical L-type lectins. The amino acid residue composition in these loops in DORN1 exhibits a higher variation than those of the templates and other L-type lectins (Fig 3 and S2 Fig).

### Homology model of the DORN1 lectin domain

**Fig 4:**
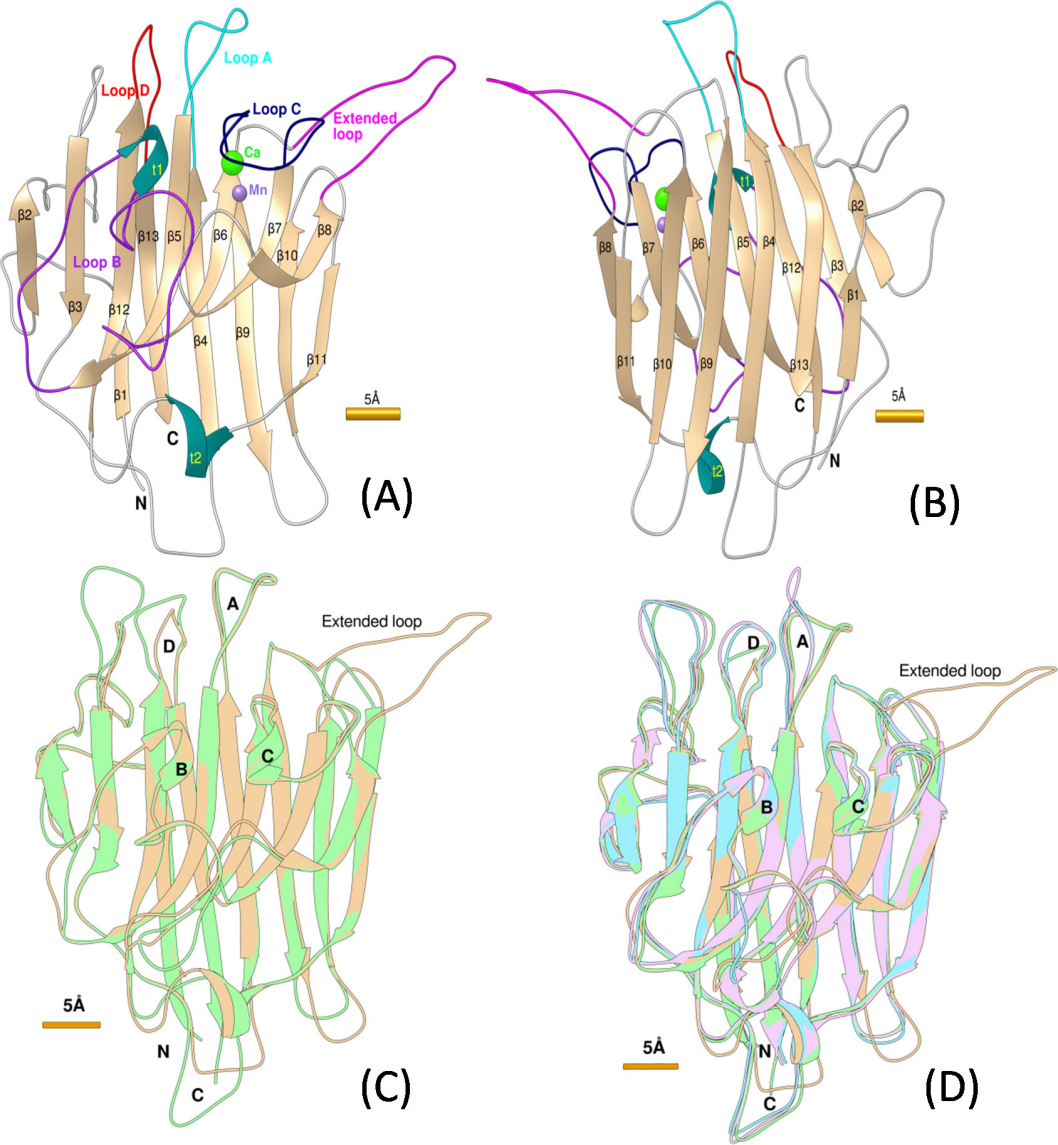
Ribbon style representations of the DORN1 models and superimposed models. (A) and (B) Backbone alpha-carbon traces of the DORN1 model with 13 defined beta strands (β1 to β13), 4 defined loops (A-cyan, B-purple, C-dark blue, and D-red), 2 β-turns (t1 and t2 in blue), 1 extended loop (magenta), and other loops in front view and back view, respectively. (C) and (D) Backbone alpha-carbon traces of the superimposed models between DORN1 (tan) with the best template (green), and with other selected templates 1BJQ (purple) and 1FAT (cyan). Yellow bars next to the figures denote relative scale of the model in Angstrom unit (1Å = 10^-10^m).

A good quality structural model of the lectin domain of DORN1 was obtained by following standard procedures in homology modeling (Fig 4A and 4B). The final model was checked for its overall stereochemical quality using the Ramachandran plot, which showed 94.9% residues in favorable regions, only 5.1% residues in allowed regions and no residues in disallowed regions (S2 Table and S3 Fig). Further analysis of the model by Verify3D revealed that 84.32% residues had an average 3D (3D structure)—1D (primary structure) traced score equal or greater than the threshold (0.2) in the 3D/1D profile comparison. After 20 picoseconds (ps) of the MD simulation, the average RMSD (Root Mean Square Difference) of the side chains of the final model was shifted 0.73Å from the initial model leading to a significant reduction of the force field energy (-229.4kcal/mol) obtained from the final model (S4A and S4B Fig). The residue DOPE score of the model was decreased after the loop refinement and MD simulation (S4C Fig). The quality of the model was also assessed by comparing the modeled structure to the crystal structure of the templates by superimposition and distant (in Å) calculation of corresponding atoms. The average RMSD of alpha-carbon trace between the homology model and the templates were 0.381Å (3IPV), 0.661Å (1FAT), and 0.96Å (1BJQ), respectively (Fig 4C and 4D). The validation and assessment steps revealed that the final model is of high quality for binding site prediction and docking studies.

### Prediction of ATP binding sites on the DORN1 lectin domain

Both the knowledge-based method and free docking method were used to predict the DORN1 ATP binding sites. The knowledge-based methods extracted sequence and/or structural features of the DORN1 lectin domain and compared them with hundreds of well characterized L-type lectins. The free docking method looked for sites which produced the smallest or lowest free binding energies calculated from the intermolecular part of the scoring conformations. Based on the prediction results, two potential ATP binding sites were identified. Interestingly, both methods yielded a common binding pocket on the DORN1 model. The location of the pocket is similar to that of the canonical carbohydrate binding site found in L-type lectins. In addition to this possible site, the free docking approach also detected another site located between loop A and the extended loop (Fig 4A).

Analysis of the common binding site predicted by both methods revealed a list of eight common residues interacting with ATP, including Gly98, His99, Thr117, Arg118, Ile143, Gly245, Thr246, and Ala247, while Asn145 was predicted only by the knowledge based method (Table 2 and S3 Table). According to the alignment (Fig 3), these residues are found on four defined loops of DORN1, except His99 lies on the β-strand just next to the loop A. In addition, scanning and comparing of residues of templates and DORN1 in the alignment also indicated that residues comprising the metal ion (Ca^2+^ and Mn^2+^) binding sites are well conserved in the DORN1 model. Similar to many L-type lectins, the metal ion binding sites are composed on five conserved residues (Glu139, Asp141, Asn145, Asp150, and His155) and an oxygen group of the main chain of Ile143, a similar residue in this position.

### Site-directed mutagenesis studies for the ATP binding site

**Fig 5:**
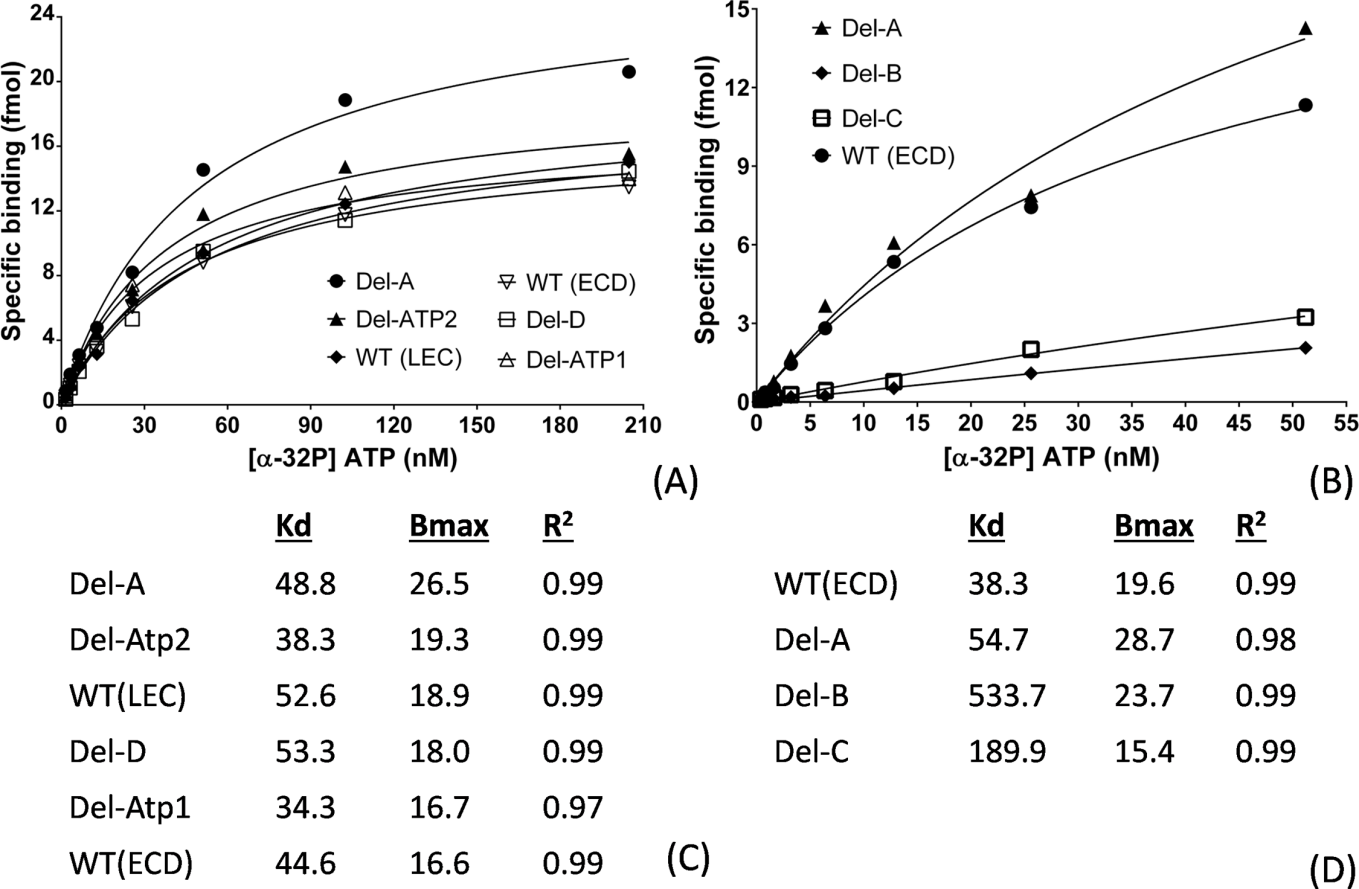
*In vitro* binding activities of ATP and DORN1 wild-types and deletion mutants. (A) and (B) Graphical representation of binding activities of ATP and DORN1 wild-types and deletion mutants *in vitro* using a non-linear regression model (see “Materials and methods” in detail). WT (LEC): lectin domain only, WT (ECD): extracellular domain, Del-A: amino acid deletion in loop A, Del-B: deletion in loop B, Del-C: deletion in loop C, Del-D: deletion in loop D and Del-ExLp1 and Del-ExLp2: deletions in the extended loop. (C) and (D) Corresponding best-fit Kd, Bmax, and R^2^ values extrapolated from the non-linear regression models of A and B, respectively.

In our initial binding site predictions, at least four out of five loops were predicted to be involved in ATP binding, including loop A, B, C, D, and the extended loop. As mentioned above, these four loops vary in size and sequence similarity between DORN1 and other L-type lectins (the alignments in Fig 3 and S2 Fig). The loop size likely does not change the core β-sheets of the monomers (Fig 4D), but it could influence their binding activity and specificity toward different ligands [25]. In order to test the model predictions, we performed site-directed mutagenesis by introducing individual deletion mutations in these five loops of the DORN1 lectin domain (Fig 2B). These deletions contain the residues predicted to directly interact with ATP (Fig 2B). As shown in Fig 5, the Del-B and Del-C mutants were significantly reduced in ATP binding activity; for example, by significant changes in the Kd values; i.e., 533.7nM and 189.9nM, respectively (Fig 5B and 5D), relative to the wild type (Kd = 38.3 nM). In contrast, the Del-A mutant resulted in little or no effect on binding affinity relative to the wild type (Fig 5C and 5D). Note that the Kd values for the wild-type protein in the Fig 5 varied somewhat (i.e., 52.6 and 38.3) likely reflecting slight differences in the independent protein preparations used. In contrast to the mutations disrupting loops B and C, there was no measurable effects on ATP binding by Del-D, Del-ExLp1and Del-ExLp2 mutants (Fig 5A and 5C). Taken together, these results indicate that for ATP ligand binding, the residues lacking in the Del-B and Del-C mutants are important and identify key residues involved in DORN1-ATP interaction. The Del-A and Del-D mutants retained some ATP binding ability, possibly due to their main chain interactions or the possibility that the deletions missed key residues for ATP binding. Meanwhile, the deletions in the extended loop did not affect the binding ability to ATP of the DORN1, indicating that the extended loop of DORN1 is not involve in ATP binding, as predicted by the DORN1 model. In conclusion, the ATP binding site of DORN1 is in a shallow pocket, which is structurally similar to the carbohydrate binding pocket in other L-type lectins, and at least loop B and loop C are essential for the binding.

### High-resolution, template and target docking

The results from redocking experiments using the AutoDockVina algorithm reveal a very similar position and conformation of GalNAc (S5A and S5B Fig) and Forssman disaccharide (S5C and S5D Fig) ligands to the template receptor (DBL or 1BJQ), as compared to the crystal structures. Interestingly, the corresponding RMSDs of the GalNAc and Forssman disaccharide ligands between the redocking and experimental structures were only 0.7Å and 0.6Å (data not shown), far below the 2Å threshold [45]. Moreover, the DBL was predicted to have a higher and competitive binding for Forssman disaccharide (-7.5kcal/mol) over the other native ligand GalNAc (-5.3kcal/mol), which was reasonable with the binding results obtained from the experimental study by Hamelryck *et al* [50]. Non-native ligands, ATP and lactose acquired a similar binding affinity (-6.7kcal/mol), but lower than that of FD and higher than that of GalNAc (A2G), respectively. Galactose (Gal) had the lowest binding affinity (-4.5kcal/mol) among the ligands used in the template docking experiments (S6 Fig). Altogether, the template docking results show that the AutoDockVina algorithm is relatively accurate and most reliable for target docking experiments of ligands to the DORN1 model.

Before docking ligands into the tested sites of the DORN1 target model, we reanalyzed the docking mode of crystal structural complexes of the 14 top ranked L-type lectins. We found two quaternary structures of L-type lectins (1BJQ and 3UJO) harboring hydrophobic molecules, adenine in a location on the back sheets of a small channel made of two monomers [50,52]. This site is not common in L-type lectins and only known for hydrophobic ligands such as adenine or adenine-derived hormones [53]. Additionally, early competitive binding studies indicated that adenine was unable to compete with ATP for binding to DORN1 [17]. Therefore, this site was eliminated from our target docking since ATP is a larger, hydrophilic molecule. The docking of ligands with all possible rotatable bonds as shown in S4 Table on the defined site of DORN1 with fixed backbone and side chain was performed, and the best binding modes with highest affinity (indicated as kcal/mol) were chosen for detailed analysis and visualization (S6 Fig).

In total, 10 docking experiments were generated for different ligand types. All ligands were successfully docked onto the predicted and tested site of the DORN1 model, and their best binding energies were summarized in S6 Fig. Interestingly, the strongest binding affinity was obtained with ATP ligand at -8.1kcal/mol, which is better than that of other nucleotides (ranging from -5.8kcal/mol for AMP to -7.4kcal/mol ADP) and much higher than that of carbohydrate substrates, e.g.,-3.9kcal/mol for galactose and -5.1kcal/mol for lactose (S6 Fig).

**Fig 6:**
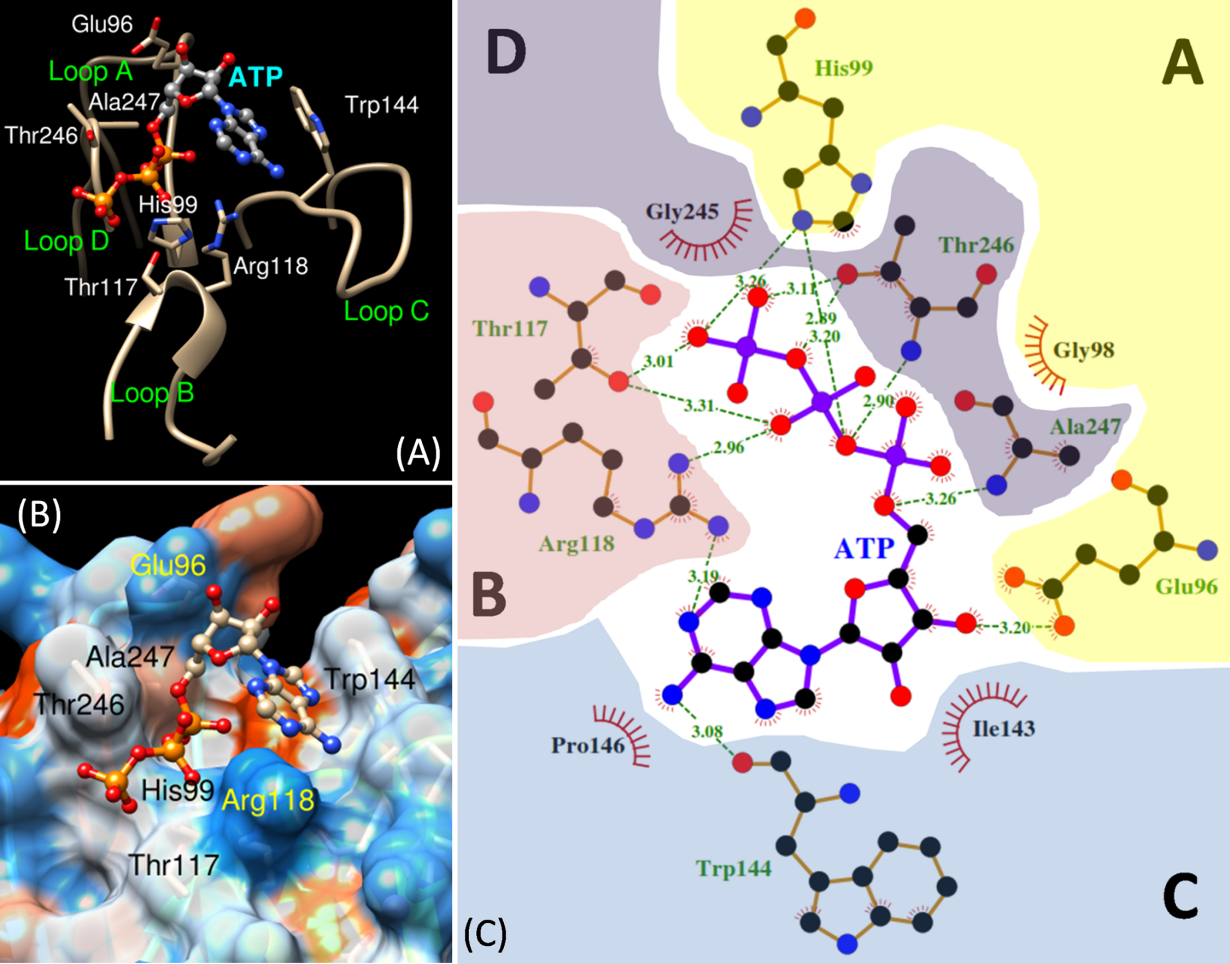
Cartoon representation of the ATP binding site and interacting residues of DORN1. (A) Close up of the DORN1 binding loops and ATP molecule in ball and stick mode, where numbered residues are those sharing H (hydrogen) bond with ATP. (B) Close up of electrostatic potential molecular surface of the DORN1 binding pocket and ATP molecule in ball and stick mode, where numbered residues are those interact with ATP via hydrogen bonds. (C) A detailed interaction map of the DORN1 and ATP docking complex, ATP resides in the middle of the map, and it is surrounded by interacting residues of 4 different loops (A to D), dashed-lines with numbers are hydrogen bonds with bond distances in angstrom sharing with atoms of ATP, where residues with a red crown denote hydrophobic interaction. Numbered residues represent their actual position of the DORN1 full-length sequence.

Detailed analysis of the best binding mode of the ATP-DORN1 docking complex revealed that ATP ligand binds to a shallow pocket surrounded by four defined loops (A, B, C and D) in the DORN1 model. The ligand interacts with residues of these loops via hydrogen bonds and hydrophobic interactions (Fig 6). The adenine ring is situated in the small cleft made of loops B and C, and the phosphate moiety is positioned in the other cleft between loops B and D. The ribose sugar stays in the middle and likely interacts with side chain of loops A and C (Fig 6A and 6B).

The binding affinity of ATP to DORN1 was derived from a network of 12 hydrogen (H) bonds and 4 hydrophobic interactions sharing between the atoms of ATP and the atoms of 11 residues located in the four defined loops of DORN1 (Fig 6C). These residues included eight residues obtained in the binding site predictions, except Asn145 (Table 2). A significant number of hydrogen bonds were observed in the phosphate group (9 H bonds) of ATP, and much higher than that of its adenine group (2 H bonds) or ribose group (1 H bond, only). These H bonds and hydrophobic interactions were made of side chains and backbone atoms of seven residues, including Glu96, His99, Thr117, Arg118, Trp144, Thr246 and Ala247. The residues involved in hydrophobic interactions were Gly98, Ile143, Pro146, and Gly245 (Fig 6C). Other ligands were shown to possess a fewer number of H bonds and hydrophobic interactions than those of ATP, especially mono- and disaccharides (S4 Table).

## Discussion

Although the L-type lectin domains are widely distributed in plants [22], their crystal structures are only available within the leguminous family, and for that reason homology modeling is currently the best complimentary approach that can build their molecular structure for studies of the molecular interaction with various ligands. Moreover, homology modeling is often a method of choice to provide reliable 3D models when a certain level of sequence and structural relationship exists among a target protein and proteins with a known crystal structure [54]. Therefore, we used these methods to construct a 3D model of the DORN1 lectin domain using homologous template structures from other, well-characterized L-type lectins.

The predicted 3D structure of the DORN1 lectin domain is folded in a 'β sandwich' architecture with 'β jelly rolls' topology, as observed in crystal structures of L-type lectins (Fig 4). The predominant structural element of the DORN1 monomer, which accounts for nearly half of its residues, organizes in two parallel β (pleated) sheets and makes a core structure of the model. One of the β-sheets contains 7 anti-parallel β-strands (β2, β3, β5, β6, β7, β8 and β12) i**n** the middle of the monomer (Fig 4A). Another β-sheet has 6 anti-parallel β-strands (β1, β4, β9, β10, β11, and β13) on one side of the subunit (Fig 4B). The remaining residues not included in the β sheets are from loops and β turns (t1 and t2) that connect all β-strands together and do not belong to regular secondary structures. Therefore, these loops create the most flexible parts of the model and, therefore, are the most difficult to predict using a modeling approach. The DORN1 model predicts an extended loop of about 13 residues inserted between β8 and β9 strands, which appears to be a unique feature of L-type lectin receptor kinases of the *Brassica* family. The extended loop contains an RGD binding motif (ASYY motif) on the tip of the β8 strand which mediates cell wall-cell membrane adhesions and has been implicated in plant resistance to the oomycete pathogen *Phytophtora* (Bouwmeester *et al*., 2011; Gouget *et al*., 2006a). However, modeling studies, as well as *in vitro* binding studies, indicate that this extended loop is not directly involved in ATP binding.

Previously, the DORN1 lectin domain was reported to have a higher degree of N-glycosylation than that of canonical L-typelectins [48]. Careful inspection of the DORN1 model, using the Chimera program for interactive visualization and analysis of molecular structures, revealed 13 Asparagine residues, of which eight residues are exposed on the surface the DORN1 lectin model (Asn37, Asn56, Asn124, Asn128, Asn181, Asn204, Asn225, and Asn232) and could serve as sites of glycosylation [48]. The other five ASN residues appear to be buried inside the protein (Asn128, Asn145, Asn154, Asn161, and Asn185) and, hence, are unlikely to be glycosylated. However, it is clear from the *in vitro* studies, using non-glycosylated, purified protein from *E*. *coli*, that glycosylation is not essential for high affinity ATP binding. Indeed, the distance of the nearest glycosylation site (Asn124) to the predicted extracellular ATP binding site of the DORN1 model is about 17Å, relatively far away from the binding site. By analogy to other, studied L-type lectins, glycosylation may play a role in protein complex formation, protein folding, sorting or transport [55,56].

Canonical binding sites for monosaccharides of L-type lectin are composed of conserved and semi-conserved residues, including Asp90 (alignment position, loop A), Gly109, Gly110 (loop B), Phe137, Asn139 (loop C), and Ala244/Leu244 (loop D) (S2 Fig). However, conservation of these residues is not universal. For example, Arcelin 1 (S2 Fig) has a truncated loop C, which appears to inactivate monosaccharide binding [28]. As mentioned, the variation in size and residue composition in the carbohydrate binding site plays an important rolein the ligand specificity and affinity of L-type lectins and has been used to define ligand types (Sharma and Surolia, 1997). In addition, most L-type lectins bind oligosaccharides at a higher affinity than monosaccharides, suggesting that additional residues interact with the ligand beside those of the monosaccharide binding site [57]. In the DORN1 model, key residues involved in carbohydrate binding are not conserved (except Asn139 in the alignment, or Asn145 in actual position of DORN1), and are replaced by His90 (His99, ATP binding), Thr109 (Thr117, ATP), Arg110 (Arg118, ATP), Ile137 and Thr244 (Thr246, ATP), respectively (S2 Fig). Therefore, DORN1 is unlikely to bind to carbohydrates, which is consistent with *in vitro* studies of direct interaction with a variety of monosaccharides [20]. Consistent with these findings, DORN1 variation from other L-type lectins in the residue composition and loop size support the higher affinity of DORN1 for ATP over other possible substrates (S6A Fig and S4 Table). Indeed, DORN1 is equipped with a set of ATP binding residues totally different from conserved carbohydrate binding residues of legume lectin templates (Fig 3 and S2 Fig). Among different nucleotides, the ATP ligand exhibits a higher competitive advantage against other nucleotides, galactose and lactose in binding to the DORN1 lectin domain (S6A Fig).

## Conclusion

In summary, our *in silico* and *in vitro* binding studies uncovered a previous unidentified ATP binding site of the DORN1 extracellular domain and confirmed that DORN1 recognizes and binds ATP with a strong binding affinity, and importantly elucidated key features of its binding site, including key residues that modulate ATP affinity. Further studies on point mutations of key binding residues could provide important insight into molecular mechanisms of interaction between extracellular ATP and DORN1 as well as its counterparts in other plant species governing growth, development and stress responses.

## Supporting Information

S1 Fig A typical design of grid coverage in 3D spaces for docking experiments of DORN1 and ATP. (A) The 3D grid space covered the DORN1 model entirely for free docking experiments, i.e. allowing ATP targets to any potential pocket on the DORN model. (B) The grid box stationed on the predicted binding site made of 4 defined loops A-D. The grid box and docking parameters were defined and set up using the MGL tool following the AutoDockVina guidelines. The parameters were adjusted for different ligands. In all docking experiments, ligands were set to have maximal number of rotatable bonds and the receptor (DORN1) was set to have rigid bonds.
(TIF)

S2 Fig Structural alignment of DORN1 and 14 top rank legume lectins. ARC1 (Arcelin 1)-*Phaseolus vulgaris* (PDB code: 1AVB), DBL-*Dolichos biflorus* (1BJQ), MAL-*Maackia amurensis* (1DBN), WBA2-*Dolichos tetragonolobus* (1F9K), DLEC2-*Phaseolus vulgaris* (1FAT), ECL-*Erythrina crista*-*galli* (1GZ9), GSI-B4-*Griffonia simplicifolia* (1HQL), LE1-*Glycine max* (1SBF), WBAI-*Dolichos tetragonolobus* (1WBF), LECA-*Pisum sativum* (2BQP), SPL-*Spatholobus parviflorus* (3IPV), DLL-II-*Dolichos lablab* (3UJO), PELA-*Platypodium elegans* (3ZYR), VML-*Vatairea macrocarpa* (4U36), and DORN1-*Arabidopsis thaliana* (P2K1). Black bars-beta strands, blue bars-beta turns, dash lines-loops or coils, *-conserved sugar binding residues of legume lectins, red dots-Mn^2+^ binding residues, blue dots-Ca^2+^ binding residues, black dots-Mn^2+^ and Ca^2+^ binding residues.
(TIF)

S3 Fig Ramachandran plots of the DORN1 model structure validation. (A) Graphical representation of alpha-carbon geometry (phi-ф, psi-ψ) and beta-carbon deviation values of the DORN1 model before refinement (B) Graphical representation of alpha-carbon geometry (phi-ф, psi-ψ) and beta-carbon deviation values of the DORN1 model after refinement. Black dots denote residues in favored regions, yellow dots represent residues in allowed regions, and red dots are residues in outlier regions, i.e. ‘bad’ residues.
(TIF)

S4 Fig Graphical representations of the DORN1 model optimization steps and quality evaluation in term of energy. (A) The atomic distance variation in terms of root mean square deviation (RMSD) between corresponding aligned atoms in angstrom scale generated during 20ps (picoseconds) of molecular dynamic simulation. (B) Total potential energy of the model side chains obtained during 20ps of molecular dynamic simulation. (C) Discrete optimized potential score (DOPE) of individual residues in the initial model (red), the optimized model (blue) and the template (green, PDB code 3IPV chain A). Note: residue positions of the template in the graph may not be in the same columns with those of the DORN1 initial and optimized models.
(TIF)

S5 Fig Graphical representation of the template binding site for GalNAc (brown) and FD (light blue) ligands. (A) Ribbon and (B) molecular surface representations of the template’s binding sites for GalNAc ligand in the crystal complex (brown) and the docking model (light blue). (C) Ribbon and (D) molecular surface representations of the template’s binding sites for Forssman disaccharide (GalNAc(α1–3)GalNAc) ligand (brown) in the crystal complex and docking model (light blue).
(TIF)

S6 Fig *In silico* binding affinity (-kcal/mol) of different ligands to DORN1 and the template DBL. Binding affinity values of nucleotides and sugars to (A) the DORN1 model and (B) the template crystal model (DBL) obtained from target docking experiments. Lac: lactose, Gal: galactose, FD: Forssman disaccharide (GalNAc(α1–3)GalNAc), A2G: N-Acetyl Galactosamine (GalNAc). FD and NAG are native ligands of 1BJQ.
(TIF)

S1 Table Fold prediction results of DORN1 L-type lectin domain using the sequence-structure homology recognition method. (DOCX)

S2 Table Stereo-chemical quality of DORN initial and refined models, and the best template structure 3IPV-chain A. (DOCX)

S3 Table Search statistics of binding sites for DORN1 using sequence and structure-based prediction tools. (DOCX)

S4 Table Number of rotatable bonds, hydrogen bonds, hydrophobic interactions and binding affinities of DORN1 and ligands. (DOCX)
