# Supplementary figures and images for: Computational Analysis of the Ligand Binding Site of the Extracellular ATP Receptor, DORN1
Source: PLoS One. 2016 Sep 1;11(9):e0161894. doi: 10.1371/journal.pone.0161894 (PMC5008829; doi:10.1371/journal.pone.0161894)

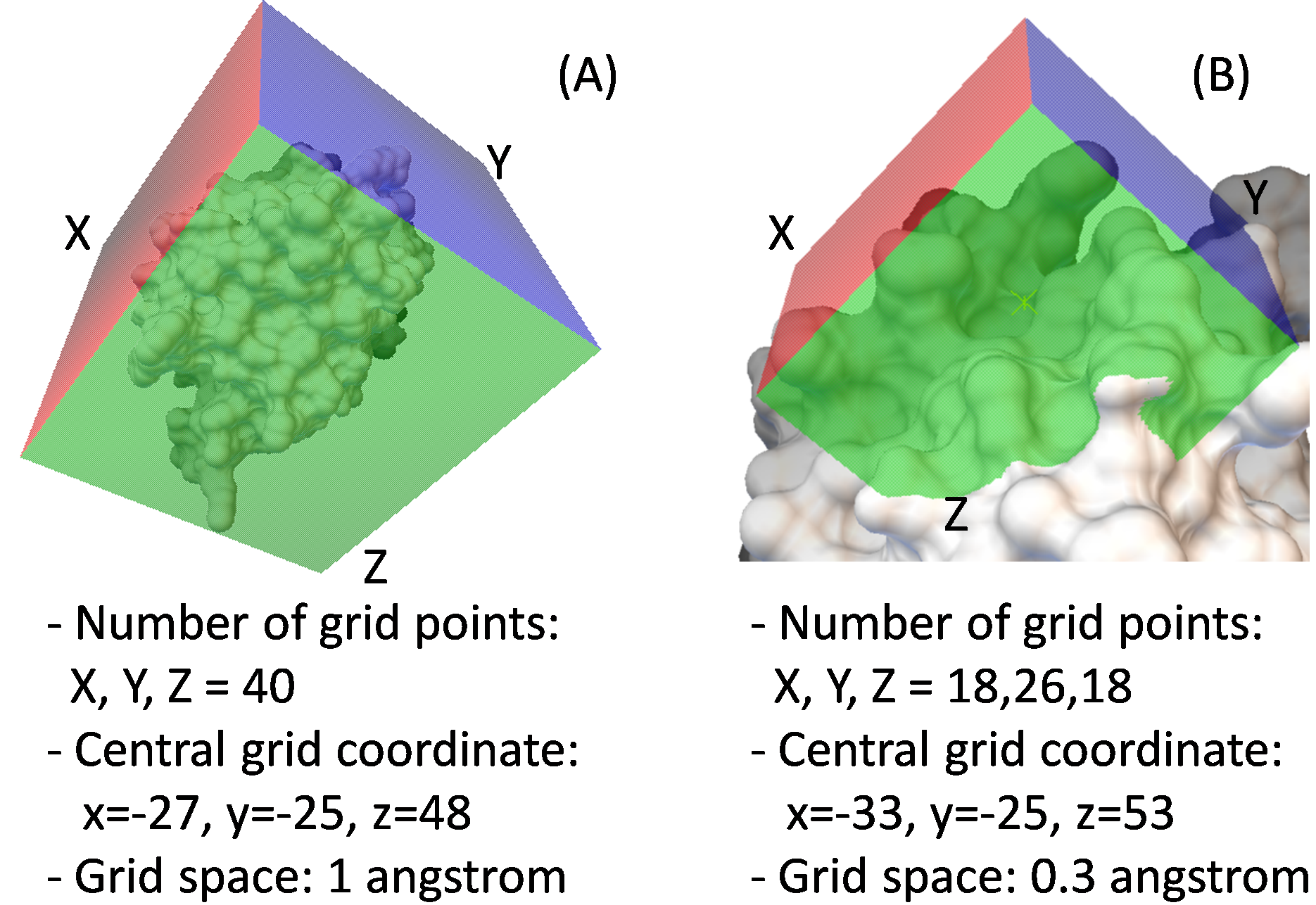

Supplement: S1 Fig — (A) The 3D grid space covered the DORN1 model entirely for free docking experiments, i.e. allowing ATP targets to any potential pocket on the DORN model. (B) The grid box stationed on the predicted binding site made of 4 defined loops A-D. The grid box and docking parameters were defined and set up using the MGL tool following the AutoDockVina guidelines. The parameters were adjusted for different ligands. In all docking experiments, ligands were set to have maximal number of rotatable bonds and the receptor (DORN1) was set to have rigid bonds. (TIF) [file pone.0161894.s001.tif]

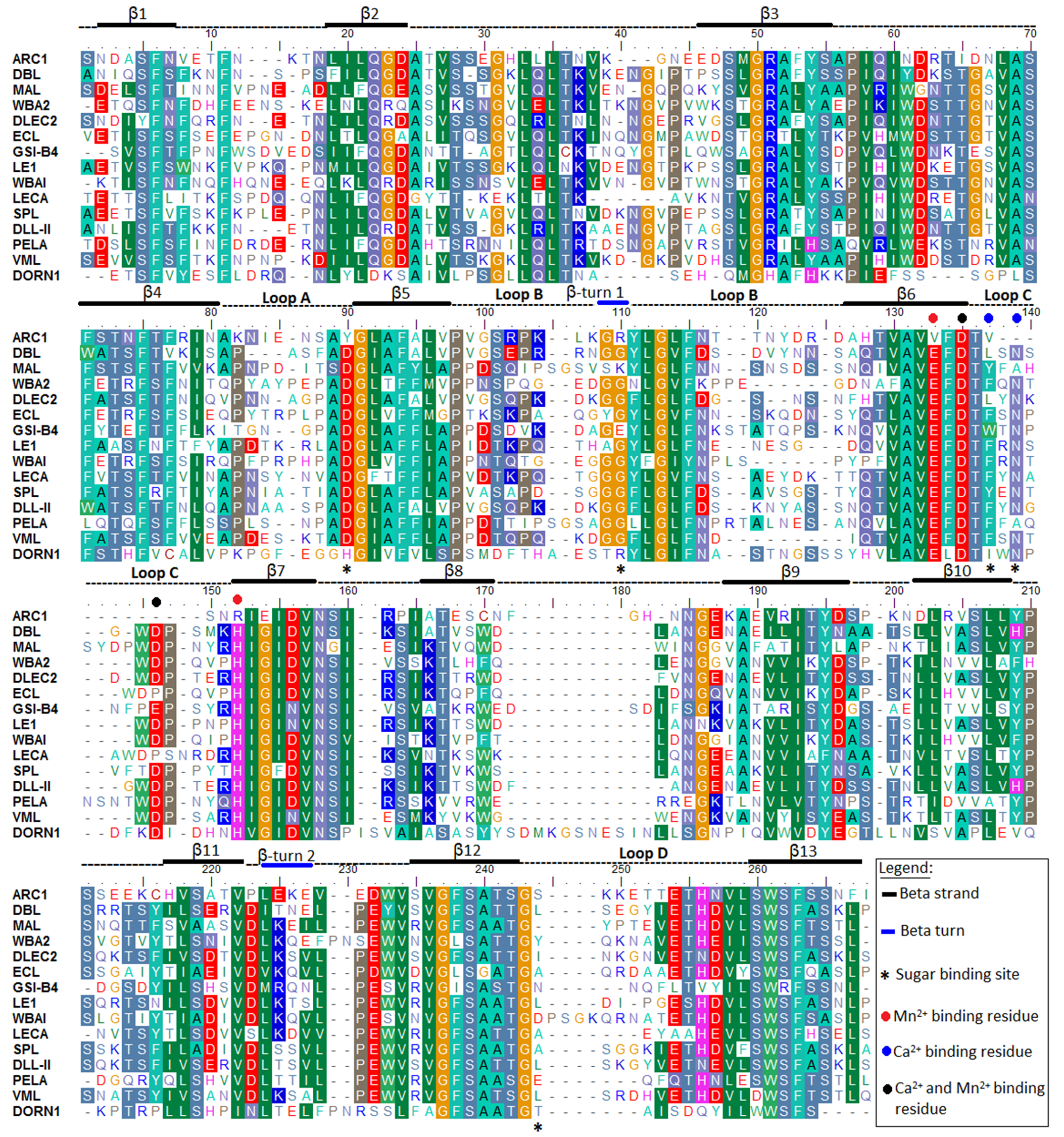

Supplement: S2 Fig — ARC1 (Arcelin 1)-Phaseolus vulgaris (PDB code: 1AVB), DBL-Dolichos biflorus (1BJQ), MAL-Maackia amurensis (1DBN), WBA2-Dolichos tetragonolobus (1F9K), DLEC2-Phaseolus vulgaris (1FAT), ECL-Erythrina crista-galli (1GZ9), GSI-B4-Griffonia simplicifolia (1HQL), LE1-Glycine max (1SBF), WBAI-Dolichos tetragonolobus (1WBF), LECA-Pisum sativum (2BQP), SPL-Spatholobus parviflorus (3IPV), DLL-II-Dolichos lablab (3UJO), PELA-Platypodium elegans (3ZYR), VML-Vatairea macrocarpa (4U36), and DORN1-Arabidopsis thaliana (P2K1). Black bars-beta strands, blue bars-beta turns, dash lines-loops or coils, *-conserved sugar binding residues of legume lectins, red dots-Mn2+ binding residues, blue dots-Ca2+ binding residues, black dots-Mn2+ and Ca2+ binding residues. (TIF) [file pone.0161894.s002.tif]

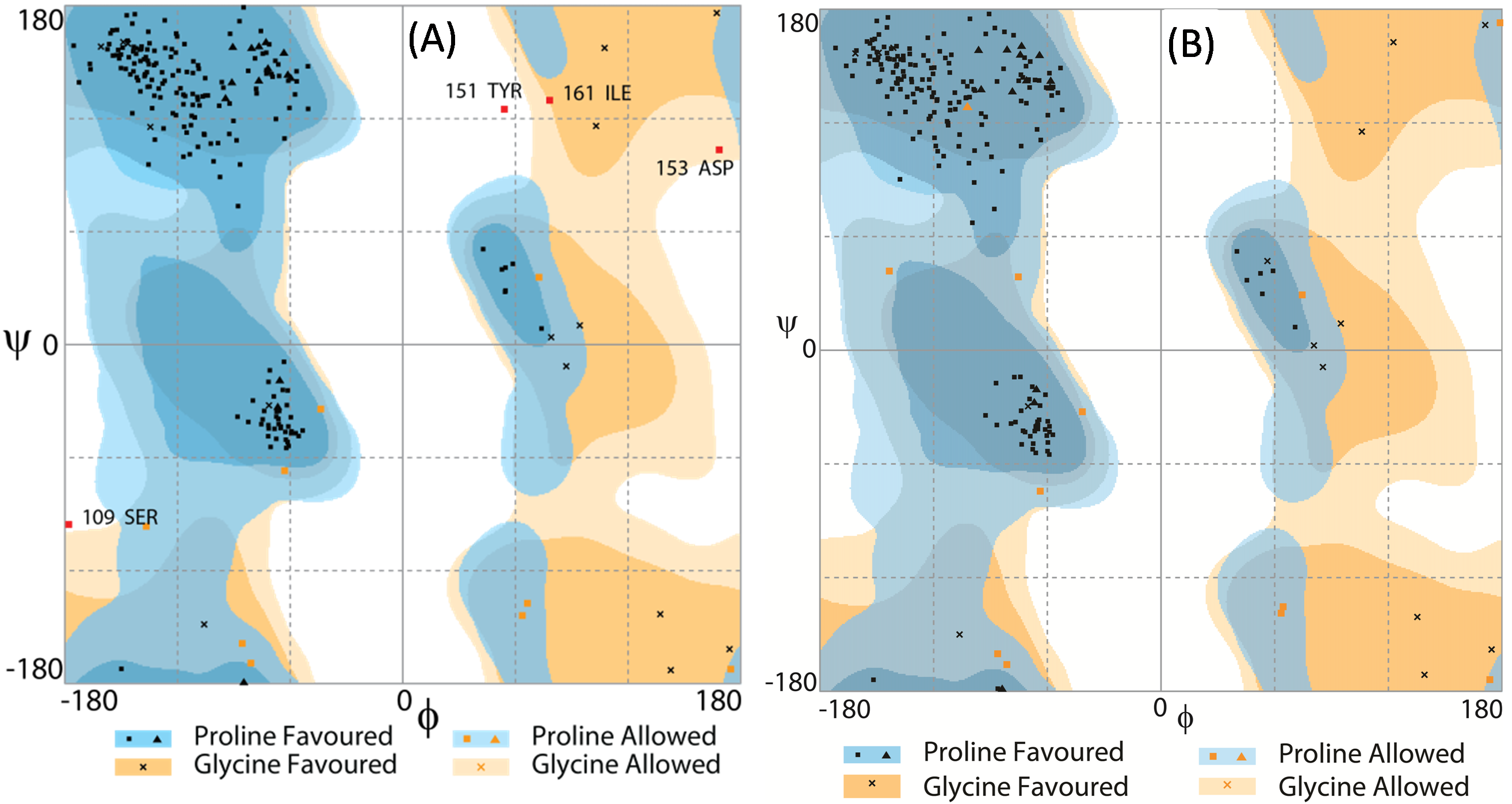

Supplement: S3 Fig — (A) Graphical representation of alpha-carbon geometry (phi-ф, psi-ψ) and beta-carbon deviation values of the DORN1 model before refinement (B) Graphical representation of alpha-carbon geometry (phi-ф, psi-ψ) and beta-carbon deviation values of the DORN1 model after refinement. Black dots denote residues in favored regions, yellow dots represent residues in allowed regions, and red dots are residues in outlier regions, i.e. ‘bad’ residues. (TIF) [file pone.0161894.s003.tif]

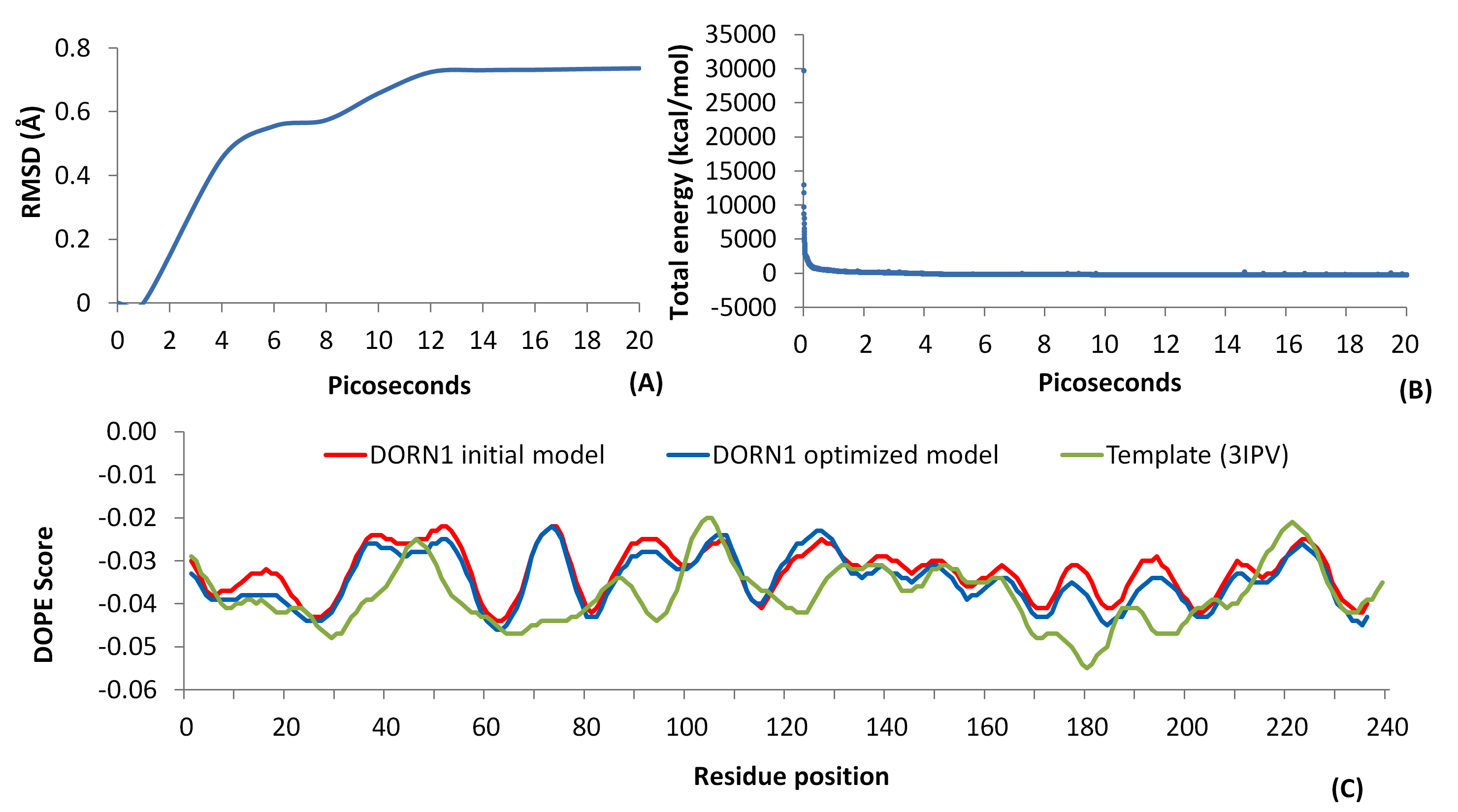

Supplement: S4 Fig — (A) The atomic distance variation in terms of root mean square deviation (RMSD) between corresponding aligned atoms in angstrom scale generated during 20ps (picoseconds) of molecular dynamic simulation. (B) Total potential energy of the model side chains obtained during 20ps of molecular dynamic simulation. (C) Discrete optimized potential score (DOPE) of individual residues in the initial model (red), the optimized model (blue) and the template (green, PDB code 3IPV chain A). Note: residue positions of the template in the graph may not be in the same columns with those of the DORN1 initial and optimized models. (TIF) [file pone.0161894.s004.tif]

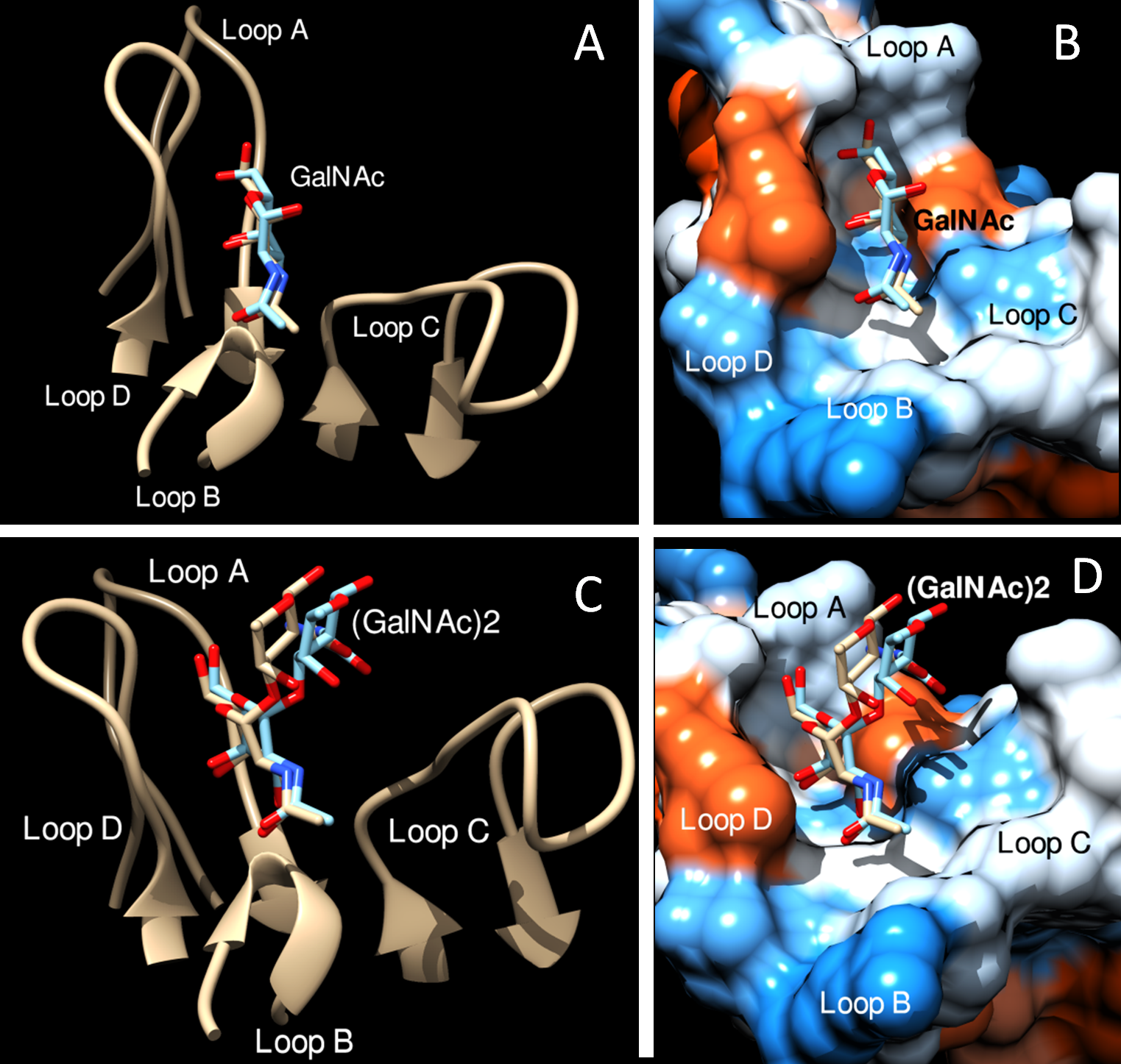

Supplement: S5 Fig — (A) Ribbon and (B) molecular surface representations of the template’s binding sites for GalNAc ligand in the crystal complex (brown) and the docking model (light blue). (C) Ribbon and (D) molecular surface representations of the template’s binding sites for Forssman disaccharide (GalNAc(α1–3)GalNAc) ligand (brown) in the crystal complex and docking model (light blue). (TIF) [file pone.0161894.s005.tif]

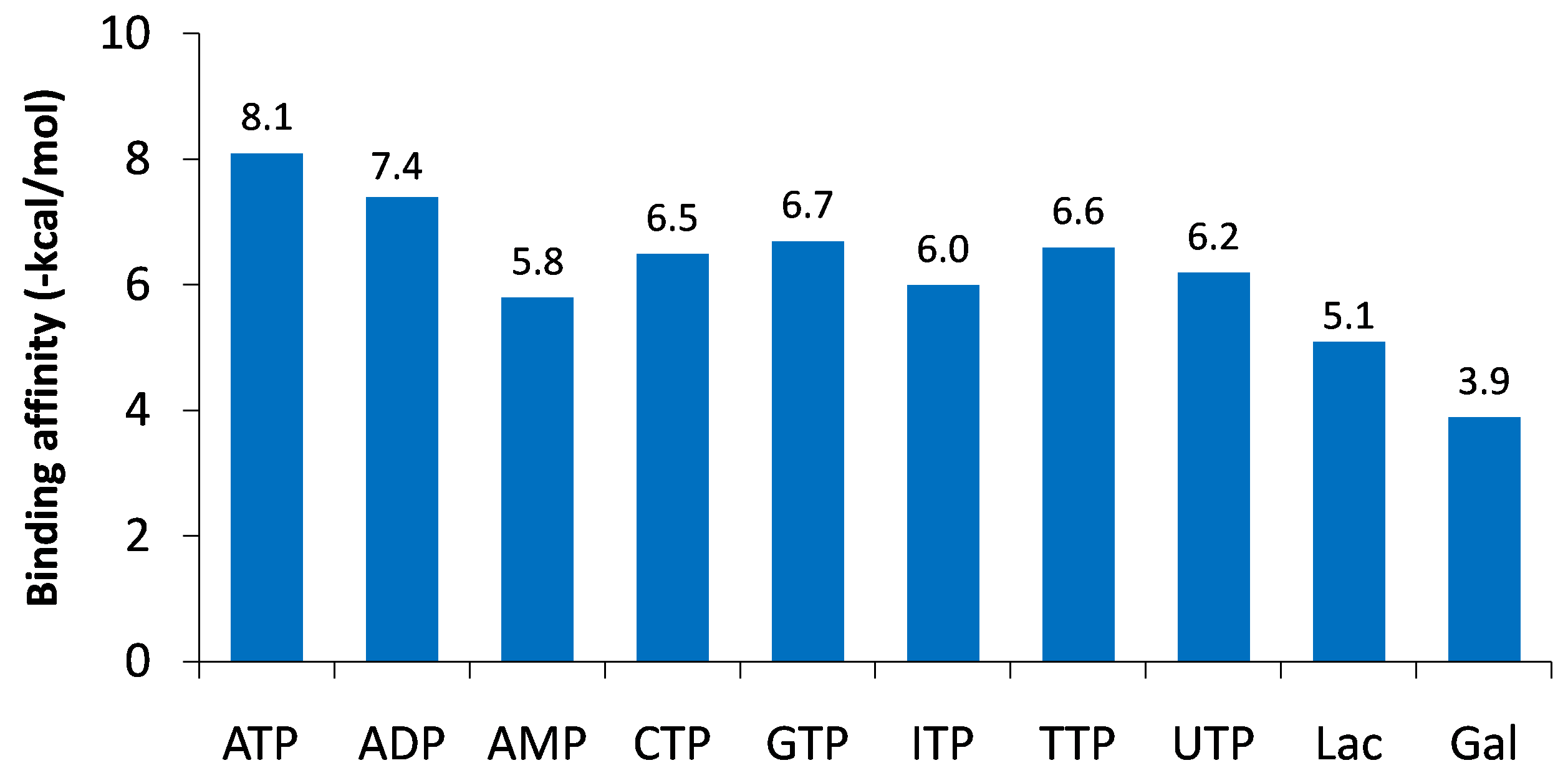

Supplement: S6 Fig — Binding affinity values of nucleotides and sugars to (A) the DORN1 model and (B) the template crystal model (DBL) obtained from target docking experiments. Lac: lactose, Gal: galactose, FD: Forssman disaccharide (GalNAc(α1–3)GalNAc), A2G: N-Acetyl Galactosamine (GalNAc). FD and NAG are native ligands of 1BJQ. (TIF) [file pone.0161894.s006.tif]
