# Supplementary material for: Computational Analysis of the Ligand Binding Site of the Extracellular ATP Receptor, DORN1
Source: PLoS One. 2016 Sep 1;11(9):e0161894. doi: 10.1371/journal.pone.0161894 (PMC5008829; doi:10.1371/journal.pone.0161894)
Supplement: S1 Table — (DOCX) [file pone.0161894.s007.docx]

**S1 Table.**

| Profile hit^1^ | Length^2^ | Normalized Z-score^3^ | Confidence level |
| --- | --- | --- | --- |
| L-type lectin | 281 | 29.06 | CERTAIN |
| p58/Ergic-53 | 223 | 11.20 | CERTAIN |
| Emp47p | 222 | 10.89 | CERTAIN |
| Lactoferrin-binding protein b | 286 | 2.87 | GUESS |
| Monomeric porin OmpG | 252 | 2.73 | GUESS |

^1^Profile name found in the HOMSTRAD database, a structural profile database used for fold recognition. ^2^The alignment length in the profile. ^3^Z-scores greater than 6 consider as having common folds.
