# Supplementary material for: Computational Analysis of the Ligand Binding Site of the Extracellular ATP Receptor, DORN1
Source: PLoS One. 2016 Sep 1;11(9):e0161894. doi: 10.1371/journal.pone.0161894 (PMC5008829; doi:10.1371/journal.pone.0161894)
Supplement: S2 Table — (DOCX) [file pone.0161894.s008.docx]

**S2 Table.**

|  | Initial model | Refined model | Template (3IPV) |
| --- | --- | --- | --- |
| In favored region | 221 (94.4%) | 222 (94.9%) | 232 (97.9%) |
| In allowed region | 9 (3.8%) | 12 (5.1%) | 4 (1.7%) |
| In outlier region | 4 (1.7%) | 0 (0.0%) | 1 (0.4%) |

Note: Number and corresponding percentage of residues in the DORN1 models and the template structure were classified in three different categories using Ramachandran plots
