# Supplementary material for: Computational Analysis of the Ligand Binding Site of the Extracellular ATP Receptor, DORN1
Source: PLoS One. 2016 Sep 1;11(9):e0161894. doi: 10.1371/journal.pone.0161894 (PMC5008829; doi:10.1371/journal.pone.0161894)
Supplement: S3 Table — (DOCX) [file pone.0161894.s009.docx]

**S3 Table.**

| Tools | Confidence score^1^ | Cluster size^2^ | Ligands^3^ | Consensus binding residues^4^ |
| --- | --- | --- | --- | --- |
| COACH | 0.67 | 301 | Glucose,  Multiple ligands | 98,99,117,118,143,  145,245,246,247 |
| COFACTOR | 0.52 | - | N-acetyl galactosamine | 99,117,118,119,  143, 145,245,246 |
| FINDSITE | 0.50 | 80 | Multiple ligands | 96,98,99,117,118,143,  145,245,246,247 |
| TM-SITE | 0.34 | 24 | Galactose, Mannose | 98,99,117,118,143,  145,245,246,247 |

^1^Score ranging from 0 - less confidence to 1 - high confidence. ^2^Cluster size: number of templates involved in prediction. ^3^Potential ligands inferred from template sequences and structures. ^4^Numbers denote residue position on the DORN1 model
