# Supplementary material for: Computational Analysis of the Ligand Binding Site of the Extracellular ATP Receptor, DORN1
Source: PLoS One. 2016 Sep 1;11(9):e0161894. doi: 10.1371/journal.pone.0161894 (PMC5008829; doi:10.1371/journal.pone.0161894)
Supplement: S4 Table — (DOCX) [file pone.0161894.s010.docx]

**S4 Table.**

| Ligands | Number of rotatable bonds^1^ | Number of H bonds | Number of hydrophobic interactions | Binding affinities  (-kcal/mol) |
| --- | --- | --- | --- | --- |
| ATP | 11 | 12 | 4 | 8.1 |
| ADP | 9 | 7 | 5 | 7.4 |
| AMP | 7 | 8 | 5 | 5.8 |
| CTP | 11 | 8 | 5 | 6.5 |
| GTP | 11 | 7 | 5 | 6.7 |
| ITP | 10 | 7 | 5 | 6.0 |
| TTP | 9 | 8 | 6 | 6.6 |
| UTP | 10 | 9 | 3 | 6.2 |
| Lactose | 12 | 6 | 6 | 5.1 |
| Galactose | 6 | 4 | 5 | 3.9 |

^1^ A maximal number of bonds are allowed to have free rotation around themselves.
